# Food systems and rural wellbeing: challenges and opportunities

**DOI:** 10.1007/s12571-021-01217-0

**Published:** 2022-02-08

**Authors:** Jim Woodhill, Avinash Kishore, Jemimah Njuki, Kristal Jones, Saher Hasnain

**Affiliations:** 1 Food Systems Group, Environmental Change Institute, University of Oxford, Oxford, UK; 2 International Food Policy Institute (IFPRI), Delhi, India; 3 International Food Policy Institute (IFPRI), Nairobi, Kenya; 4 JG Research and Evaluation, Bozeman, USA; 5 Food Systems Group, Environmental Change Institute, University of Oxford, Oxford, UK

**Keywords:** Food systems, Rural poverty, Small-scale farming, Livelihoods

## Abstract

The future wellbeing of billions of rural people is interconnected with transforming food systems for equity, nutrition, environmental sustainability, and resilience. This article tackles three blind spots in the understanding of rural poverty and vulnerability: the narrow focus on extreme poverty and hunger that hides a much wider set of inequalities and vulnerabilities, insufficient recognition of the diversity of rural households, and an inadequate appreciation of the impact of rapid structural changes in markets, the physical environment, and the political economic context. A better understanding of these areas is necessary for imagining a new policy landscape that can align progress on rural poverty alleviation with a wider transformation of food systems. The article provides a framework for assessing the dynamics of rural wellbeing and food systems change. It looks at the viability of small-scale farming and the diversification of livelihood options needed to overcome rural poverty and inequality. The analysis suggests that the future prosperity of rural areas will depend on policy reforms to address market failures in the food system, which currently work against equity, good nutrition and sustainability. Investments will also be needed to enable rural economies to capture greater value from the food system, particularly in the midstream of food distribution, processing and services. The likely future scale and nature of rural poverty and inequality is such that improved social protection and humanitarian relief schemes that support those in crisis or being left behind will still be essential.

## Introduction

The future wellbeing of billions of rural people hinges on transforming food systems to improve equity, nutrition, environmental sustainability, and resilience. This article addresses what we believe are three blind spots in the collective understanding of rural poverty and vulnerability in the global South. First is a narrow yet widespread focus on extreme poverty and hunger that hides a wider set of inequalities and vulnerabilities. The second is insufficient recognition of the diversity of rural households with many of their livelihoods increasingly depending on a mix of on- and off-farm income sources alongside food production for self-consumption. The third is an inadequate appreciation of the impact of rapid structural changes in markets, the physical environment and the political economic context. A better understanding of these areas is necessary to design new policies that align progress on rural poverty alleviation with a wider transformation of food systems.

The purpose of this article is to provide a conceptual framing of the linkages between rural wellbeing and food systems transformation and to present data that illustrates the scale and nature of the challenges faced by policy makers. While elements of this analysis are established and understood in the academic literature, in our view, significant gaps remain in integrating and synthesising such understanding in ways that are accessible to policy makers. We argue the need for a wider and more nuanced debate about the linkages between food systems, rural poverty, and small-scale agriculture.

In the low- and middle-income countries, nearly 3.4 billion people still live in rural areas (UNDESA, 2019) and most still depend to varying degrees on agriculture and food systems for their livelihoods. Critically, this rural population includes the large majority of those who are extremely and moderately poor and/or undernourished (UNDESA, 2021). Despite rapid urbanisation, large or increasing rural populations will be a reality for most low- and middle-income countries for the foreseeable future (ibid). Meeting the Sustainable Development Goals, in particular SDG One (Poverty) and SDG Two (Hunger), and a longer-term agenda of “leaving no-one behind” will require properly addressing the linkages between rural people’s wellbeing and food systems (FAO, 2017b).

In this chapter we use the concept of rural wellbeing (OECD, 2020a) to bring a holistic and integrated perspective of how changing food systems influence rural people’s lives and security. The COVID-19 pandemic and the locust outbreaks across East Africa highlight how vulnerable rural people are to various forms of shocks to food and economic systems. Climate change driven extreme weather events, natural disasters and pest and disease outbreaks affecting both humans and agriculture are likely to increase, possibly dramatically, over the coming decades (Calicioglu et al., 2019; Gregory et al., 2009; Marvin et al., 2013). These events have the potential to seriously affect vast numbers of rural people, hampering efforts to reduce existing poverty, pushing people back into poverty and potentially creating large scale humanitarian crises (Islam & Winkel, 2017; Nicoson et al., 2019). Creating more resilient food systems is central to buffering against these shocks and vulnerabilities affecting the rural poor.

Poor rural people have opportunities and face risks as food systems change and from the implications of the call for a food systems transformation. On the one hand, growing demand for food in general and for higher value and more nutritious food products can be a substantial driver of rural economic development (Pawlak & Kołodziejczak, 2020). On the other there is the risk that these economic opportunities will be captured by a minority. To avoid this, governments will need policies to ensure that opportunities result in inclusive rural economic development. There is also the risk that solutions for the current nutritional and environmental failings of the food system come at a cost rather than a benefit to the rural poor, for example through standards that smaller producers find difficult to meet.

This article explores three important dimensions of the relationship between food systems and rural wellbeing: livelihoods, nutrition and vulnerability. Livelihood refers to the resources, financial and other, that people are able to attain to meet their needs such as food, health, education, housing and leisure (UNDP, 2010). Nutrition refers to people’s overall food and nutrition security (Hwalla et al., 2016). Vulnerability refers to people’s capacity (or lack thereof) to sustain their wellbeing in the face of risks and shocks be they from climate change, declining natural resources, disease outbreaks, market instability or political instability (O’Brien et al., 2007; Parry et al., 2007; Porter et al., 2014).

This article argues that rural wellbeing must be understood against the backdrop of diversifying patterns of employment and income. It makes little sense to speak of the ‘rural poor’ or ‘small-scale farmers’ as a homogenous group. Different strategies and policies are needed, tailored to the specific needs of different groups living in different contexts. This article explicitly focuses on rural households rather than just farming households. There is no doubt that small-scale family farming is critical to the future of food systems and rural wellbeing (P. Hazell et al., 2010; HLPE, 2013; Hwalla et al., 2016; IFPRI, 2020; Wiggins et al., 2010). However, it is also apparent that very large numbers of small-scale farmers will be unable to make a viable living from farming alone (Fan & Rue, 2020; Gneiting, 2018). A much more nuanced understanding of the diversity of small-scale farming is needed, along with a more integrated perspective of on and off-farm livelihood options.

Food systems change and rural wellbeing must be seen through an explicit gender lens. Gender inequalities are critically important in terms of poverty, nutrition, and vulnerability. The empowerment of women and girls, as well as rural youth, through economic opportunity, education and inclusion in decision-making at all levels is essential to any strategy for improving rural wellbeing (FAO, 2020b). Despite this, less than a quarter of the indicators required to monitor gender across the 2030 Agenda are available in a gender-disaggregated way (Commission on the Status of Women, 2018). In this article, we raise the challenges faced uniquely or disproportionately by women to achieving rural wellbeing to highlight the gendered dimensions of food system and rural wellbeing.

The article first provides a conceptual framing of the linkages between food systems and rural wellbeing. It then examines the diversification of rural livelihoods and the changing role of farming in household incomes. We take a closer look at the trajectories of rural wellbeing in terms of livelihoods, nutrition and vulnerability. This leads to a deeper exploration of dynamics between rural wellbeing and food systems change and concludes with the implications for policy.

We take a global perspective but give more attention to regions where there are higher levels of rural poverty in particular sub-Saharan Africa and South Asia. While there is much commonality in the underlying dynamics of rural poverty and food systems across all regions and countries, we also fully acknowledge the substantial differences between regions and countries and argue the need for better data to develop a comprehensive mapping of food system and rural poverty dynamics at the national scale.

## Conceptual framing—taking a systems perspective

**Fig. 1:**
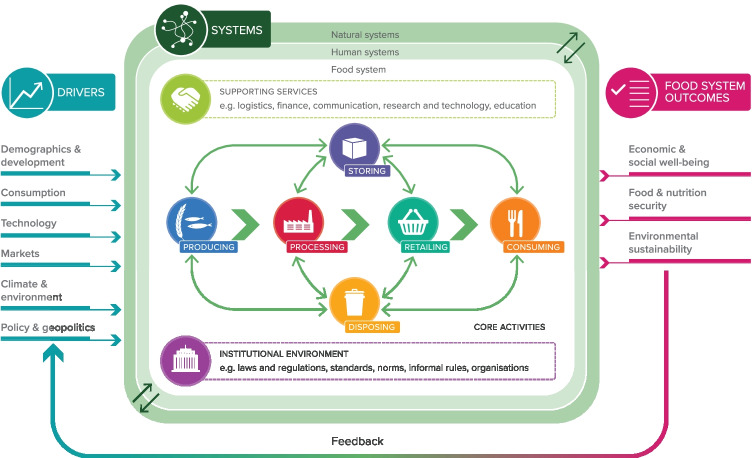
Conceptual model of a food system. Source: Woodhill (2019)

Recent years have seen issues of food security, nutrition and agriculture merged into a wider narrative of food systems. This is not just semantics. The shift signals a more holistic view of nutrition and its links with health, the interlinkages been food production climate and environment and the critical role that food systems play in employment and the economy. The food system, as understood in this article, is illustrated in Fig. 1 (Woodhill, 2019), drawing on (Ingram & Zurek, 2011; Van Berkum & Ruben, 2018).

A set of food system mega-trends have emerged. Demand will substantially increase and change due to population growth, urbanisation, and the demands of a growing middle class (FAO, 2017a, b). At the same time, the world faces a health crisis from the ‘triple burden’ of undernutrition, micronutrient deficiencies and overnutrition (FAO, 2020a). Food system activities will continue to contribute significantly to greenhouse gas emissions, and climate change risks negative impacts for food production and food security (Springmann et al., 2018; Vermeulen et al., 2012). Furthermore, how food is produced means we are overshooting the earth’s capacity to sustainably meet demand (Springmann et al., 2018; Willett et al., 2019).

**Fig. 2:**
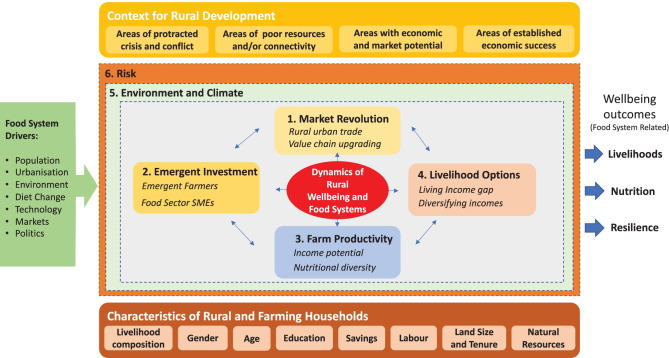
Dimensions for exploring the linkages between rural wellbeing and food systems. Source: Adapted from Woodhill et al. (2020)

Figure 2 connects this wider conception of food systems to the dynamics of rural wellbeing. Our focus is on the degree to which food systems are delivering wellbeing for rural people – or not, in terms of livelihoods, nutrition and resilience (see outcomes at the right of the diagram). The wider set of food system drivers discussed above are on the left. At the centre of the diagram are four factors influencing the trajectories of rural well-being: changing food markets (1), investments patterns (2), farm productivity (3) and livelihood options (4) which result from the dynamics of the other three factors. These factors are influenced by a wider context of environmental and climatic conditions, and a set of risks related to these conditions as well as to other risks including market fluctuations, pests and disease or personal misadventure.

## Beyond just farming

Historically, most rural people were farmers, with most of their income from farming. This led to rural development and poverty alleviation programs that focused heavily on agricultural productivity, agricultural market development and small-scale farmer commercialisation. Linked to this was a general development narrative of agricultural productivity growth driving wider economic development that eventually leads to jobs being created in the wider economy (Mellor, 2017). Theoretically, this results in a substantial drop in employment in the agricultural sector (Headey et al., 2010) as people move to higher paying and (sometimes) less arduous opportunities in manufacturing and services, resulting in the very low levels of farm employment seen in most OECD economies.

For many low- and middle-income countries this shift out of agriculture has not been as simple or as fast as theory might have predicted. Population growth, limited jobs in other sectors and people’s tendency to hold onto land has meant continued high levels of employment in agriculture and an increasing rather than decreasing number of small-scale farms (Fan & Rue, 2020; Hazell, 2015; Nolte & Ostermeier, 2017). However, this has also been accompanied by a significant diversification of farming household income, driven by both opportunity and necessity (Loison & Bignebat, 2017; Reardon et al., 2006).

The total rural population for low- and middle-income countries is approximately 3.4 billion (UNDESA, 2019). With approximately 450 million small-scale farms and taking account of family sizes, these countries have some 2–3 billion people living in households that farm, i.e., approximately 60% of the rural population. However, the incomes of rural households are diversifying dramatically through on- and off-farm employment, remittances, non-farm micro enterprises, trading and social protection payments (FAO, 2017b, pp. 79–83). Country-level census data and data from smaller case studies suggest, for example, that agriculture currently contributes roughly 40% of rural household income in India (Pingali et al., 2019), 33% in Bangladesh (Ahmed et al., 2015), and 82% in high-agricultural potential rural areas in Ethiopia (Bachewe et al., 2020). Increasingly the reality is not one of small-scale farming households, but of rural households who also farm. In India, for example, 88% of farming households also have some non-farm income (Pingali et al., 2019). Similarly, Roy and Basu (2020) find that in coastal areas of Bangladesh at least 25% of very small-scale (owning less than 1 ha) farmers also have off-farm income. However, there is a lack of comprehensive country-level data on how the livelihoods of rural households are changing, and on the distribution of employment and income across on and off-farm activities.

Income diversification has significant implications. Many rural households are becoming less dependent on their agricultural production and farm income, with this becoming just part of their overall livelihood strategy. This means what becomes important is the return to labour from farming and how this compares with to other income earning opportunities. Having a very small plot of land is not necessarily a problem if it is a complement to other sources of income, provided it gives a competitive return to labour. These changes also have significant gender implications in terms of the balance of farm work being undertaken by women, their role in off-farm enterprise and employment, and the inequalities they face as economic actors.

Currently the only disaggregated data on small-scale agriculture is in relation to farm size. Lowder et al., (2016, 2019) conducted a comprehensive review on farm numbers and farm size distribution. They conclude that globally there are at least 540 million farms, of these 90% are family farms and some 447 million farms, or 84% are < 2 ha and operate 12% of agricultural land. Drawing on this analysis and food and nutrient supply by farm scale (Herrero et al., 2017; Ricciardi et al., 2018), a categorization of farms by land size and food production is categorised in Table 1 (adapted from Woodhill et al., 2020). Data availability, differences between countries and methodological challenges mean this analysis is indicative, pointing to larger trends that need further investigation. However, the overall picture aligns with detailed field observations in Africa and India (Giller et al., 2021).

**Table 1:**
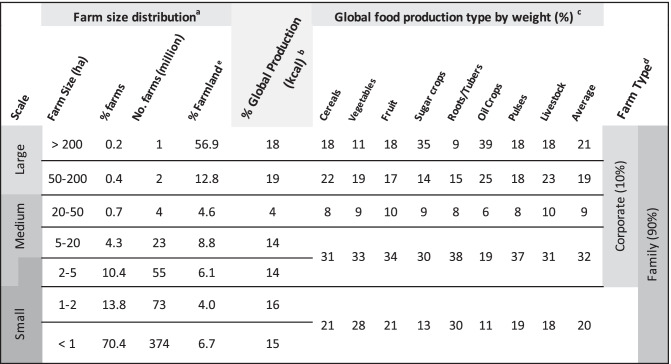
Indicative characteristics of farm numbers, area farmed and food production related to farm size. Source: Modified from Woodhill et al. (2020) based on data from Lowder et al. (2019), Ricciardi et al. (2018) and Herrero et al. (2017): ^a^ data from Lowder et al. (2019), Table A2—estimates based on 129 countries; ^b^ data from Ricciardi et al. (2018), values estimated from Fig. 2H—based on 55 countries; ^c^ data from Herrero et al. (2017), values estimated from Fig. 1—based on 161 countries; ^d^ data from Lowder et al. (2019); ^e^ data from Lowder et al. (2019) show that farms of < 2 ha use around 11% of farmland while Ricciardi et al. (2018) estimate this to be about 24%

**Fig. 3:**
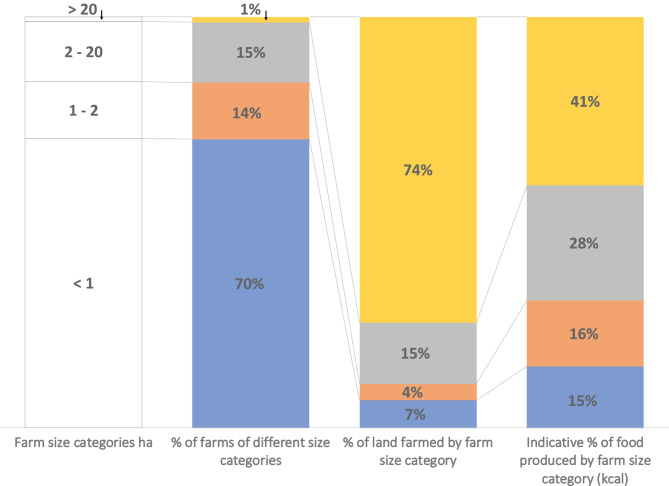
Indicative relationship between farm size, area of land farmed, and food produced. Source: data from Table 1

There are two important observations from this data (Fig. 3). First is the very large number of very small-scale farms. Of all small-scale farms < 2 ha, 86%, or 374 million, are less than 1 ha, with many much smaller still. This group constitutes 70% of all farms globally. The reality for farmers growing staple crops – or traditional cash crops such as coffee and cocoa – on these small areas of land, is the difficulty of making a living, given often low productivity and current market prices. Many poor rural households are net purchasers of food (Aksoy & Isik-Dikmelik, 2008). Combined with an increasing need for income to cover the costs of housing, education, transport and health, this makes off-farm income a necessity for many small-scale farming households.

The second observation is that most food is not produced by this very large number of very small farms that together farm a relatively small area of land. An oft-used justification for supporting small-scale agriculture is that small-scale farmers produce 70% food consumed in low- and middle-income countries (Ricciardi et al., 2018). While it may be true that all smaller-scale farmers of less than 20 ha produce this 70% (see Fig. 3), this generalisation hides the reality that on average the bulk of this 70% of food is likely produced by a smaller number of larger-scale small-scale farmers. This suggests a dualism in small-scale agriculture between very large numbers of very small-scale farmers who do not produce a great deal of food and a smaller number of larger small-scale farmers who produce most of the food. The food this larger group of very small-scale farmers produce is critical for their own income and food and nutrition security, and for localised markets, but less so for meeting the growing demands of urban populations.

This emerging dualism of small-scale agriculture means that it is important not to conflate the challenges of tackling the poverty and malnutrition of small-scale farming families with the challenge of meeting growing food supply demands for urban populations (Gassner et al., 2019). If a smaller group of farmers who have more substantial assets are already meeting the bulk of food demand, the market options for the very large numbers with much fewer assets are limited. While there is no doubt that the challenges of tackling rural poverty and ensuring domestic and global food security overlap there is a need for sharper analysis of the degree to which agricultural production on its own can lift very small-scale producers out of poverty or ensure their food and nutrition security.

**Fig. 4:**
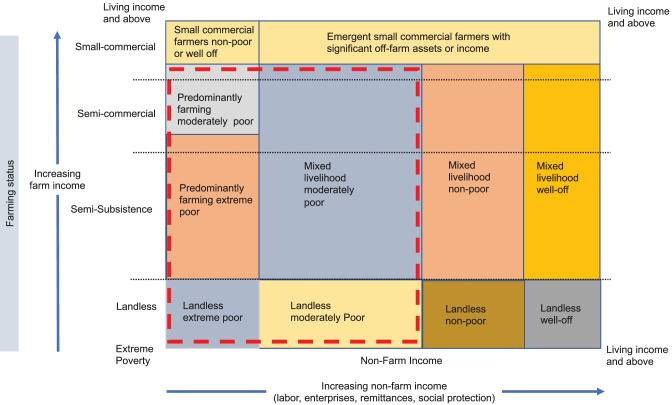
Indicative distribution of different household livelihood mix relative to degree of farm and non-farm income. Source: authors own elaboration

The diversification of household incomes and the relationship to different degrees of farm commercialisation and non-farm income is illustrated in Fig. 4. This categorisation builds on previous authors categorisation of farming types (Berdegué & Escobar, 2002; Mangnus & Metz, 2019; Vorley, 2002; Woodhill et al., 2020) to take better account of non-farm income. The relative sizes of the boxes of different categories will vary by locality and country but have been calibrated to give an indicative impression of the global situation. The extreme poor tend to be either very small-scale farmers with minimal non-farm income or landless who have limited sources of income. By far most rural households (see the red dashed box) remain extremely or moderately poor, and an increasing majority of these have a mixed livelihood with income from farm and non-farm sources. The number of small-scale commercial farmers able to make a living income with minimal off-farm income is relatively low. While precise figures do not exist, most experts with hands-on knowledge do not assume this to be much above 10% of all small-scale farmers. There is also a growing group of emergent small-commercial farmers who are salaried urban workers investing back into agriculture and who are able to support this with substantial off-farm income and assets (Jayne et al., 2016a, b). In many locations, a growing group of non-poor and well-off households are important to the rural economy. A more nuanced understanding of this diversity in rural households and how it is changing is necessary for the development of strategies and polies to optimise an inclusive transformation of food systems.

## Trends in rural wellbeing

This section considers trends in rural wellbeing from the perspectives of livelihood, nutrition and vulnerability. The status of rural people’s wellbeing is a mixed and often contradictory picture with evidence to mount both optimistic and pessimistic perspectives. It is unquestionable that over the last decades vast numbers of people have been lifted out of extreme rural poverty and hunger through agricultural development and wider economic growth (Birner & Resnick, 2010; Fan & Rue, 2020). In many countries, rural villages and towns are unrecognisable from just a decade or two ago in terms of their economic activity, wealth, infrastructure, and rural–urban linkages. However, this uplifting has been far from universal (UNDESA, 2021). There are very significant differences across regions and countries, as well as between genders and different ethnic groups. There is no doubt that very large numbers of people at the bottom of the economic pyramid, and in marginal and strife-torn areas, are being left behind (Fan & Rue, 2020; IFPRI, 2020).

The view of progress is shaped by the metrics used. The dominant metrics for assessing rural wellbeing have been the percentage of people living in extreme poverty and the percentage of child stunting. These metrics, while important, overshadow the scale of moderate poverty, the escalating scale of over-nutrition and nutrient deficiencies, and rural people’s vulnerability to shocks. This section takes a broader perspective on assessing rural wellbeing.

Table 2 provides a snapshot of the most recent data available of rural wellbeing metrics, for illustrative countries and regions. The data highlights significant differences across these regions and countries, which reinforces the need for disaggregated analysis and responses tailored to the needs of specific situations. It shows the vast numbers of moderately and extreme poor in sub-Saharan Africa and South Asia relative to the rest of the world, and the dominance of agricultural employment for vast numbers of rural people. It also illustrates the nutritional issue of continued high levels of child stunting and the significant uptick in obesity for the more urbanised LAC and MENA regions.

**Table 2:** Metrics on rural wellbeing for example countries and regions. Source: ^1^ World Bank, 2020a; ^2^ World Bank, 2020b; ^3^ World Bank, 2020c; ^4^ ILO, 2020; ^5^ Lowder et al., 2019; ^6^ Castañeda & Newhouse, 2016; ^7^ World Bank, 2020d; ^8^ FAO, 2020a; ^9^ WHO, 2020a, b

Indicator	Countries						Regions				
	DRC	ETH	GHA	BGL	IND	PAK	SSA	SA	EA	LAC	MENA
Rural population: % of population (million individuals)^1^	55 47.7	79 88.3	43 13.2	63 102.1	66 895.0	63 136.7	59 655.5	66 1,203.7	43 909.3	19 118.5	38 148.4
Rural poor (3.20): % of total population (million individuals)^2^	45 30.0	56 50.7	30 6.6	58 85.3	44 553.1	30 55.0	49 469.7	44 804.3	18 410.3	2 12.5	2 9.6
Extreme poverty in rural areas: % of rural population (million individuals)^3^	No data	35 30.8	24 3.2	18 18.3	25 222.0	5.5 7.5	45 297.2	27 329.0	15 136.8	No data	No data
Rural employment in agriculture: % of rural employment (million individuals)^4^	No data	71 31.4	37 3.1	31 23.8	35 190.3	33 24.5	72 182.4	56 257.4	48* 86.6	47 29.4	34 20.3
Small-scale farms (< 1 ha): % of total farms (million farms)^5^	85 3.8	59 8.5	No data	84 12.8	68 99.9	36 2.4	52 28.5	69 120.0	86 211.3	16 3.1	56 6.8
Small-scale farms (1–2 ha): % of total farms (million farms)^5^	10 0.5	25 3.6	No data	7 1.1	18 25.8	22 1.4	19 12.0	17 29.1	8 19.2	20 3.9	10 1.2
Extreme poor working in agriculture: % of extreme poor (million individuals)^6^	No data	No data	No data	No data	No data	No data	76 498.2	56 674.1	49 445.5	68 80.6	No data
Moderate or severe food insecurity: % of population (million individuals)^7^	No data	58 63.2	51 15.2	32 50.8	32^a^ 426.5	No data	56 577.5	33 633.3	19* 126.1	32 203.7	30 153.4
Undernourishment: % of population (million individuals)^8^	No data	20 21.5	7 1.9	13 21.0	14 189.2	12 26.1	22 224.3	13 254.7	10* 64.1	7 45.9	9 45.6
Rural stunting: % of children under five^9^	47	40	22	31	41	48	31	32	25	8	15
Rural obesity: % of non-pregnant women or adults^9^	1.2	0.4	9	3	3	11	9	5	7	24	27
Rural electricity: % of population lacking access (million individuals)^7^	No data	67 59.5	33 4.3	22 22.2	7 63.3	46 62.3	No data	No data	No data	No data	No data
Rural water: % of population lacking access (million individuals)^7^	77 36.8	69 60.9	32 4.3	3 3.3	9 80.6	10 13.8	54 356.4	9 114.2	14 129.1	13 15.5	13 18.8

### Livelihoods

This section discusses data on projected poverty levels, rates of poverty in rural areas, the extent to which poorer people are employed in the agriculture sector, gender inequalities and youth unemployment. The current international *extreme* poverty line is $1.90 a day. *Moderate* poverty is the population living under the international poverty line of $3.20 a day, typical for lower- and middle-income countries (this is an update from the previous level of $3.10 a day and we use both in the analysis as the current estimates at $3.20 have not been disaggregated by rural and urban locations). For poverty in general we use the international poverty line of $5.50 a day. Current poverty trends underscore the need for continued focus on rural poverty and inequality (UNDESA, 2021; United Nations, 2019). We argue that development efforts should focus on creating a living income for people. This is the income that people need, in their circumstances, to afford a healthy diet, housing, education, and health care, and to meet other social and family needs and responsibilities (Giller et al., 2021; Gneiting, 2018; van de Ven et al., n.d.). For most rural households in most locations, the extreme and moderate poverty rate falls very far short of a decent living income and what people need to realise their life ambitions. Extreme poverty and hunger remains a critical concern still affecting 600 to 700 million people or nearly 10% of the world population, with COVID-19 likely to increase numbers by 100 million (World Bank, 2020f). However, the even bigger challenges for future rural development efforts are the vast numbers of rural people living in moderate poverty, a rapidly growing rural youth population in many countries, and continued gender inequalities.

**Fig. 5:**
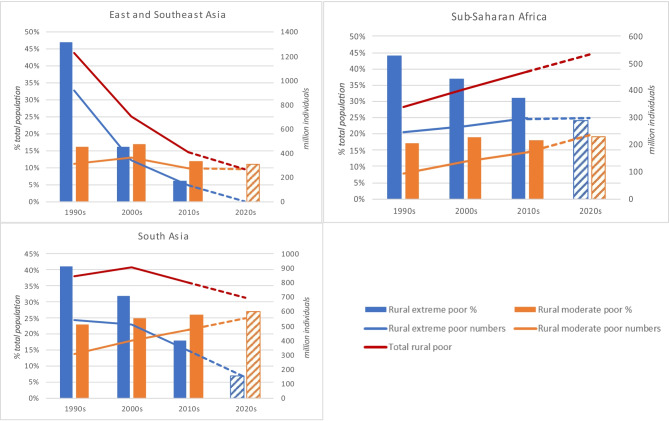
Trends in extreme and moderate poverty levels by region. Source Data from (De La O Campos et al., 2018), population estimates taken for the mid-point year of each decade (1995, 2005, 2015, 2025) from (World Bank, 2020e) and projected trajectories calculated as a simple linear estimate based on the three time points provided

For all regions except Sub-Saharan Africa, extreme poverty levels are decreasing. By around 2050, almost all extreme poverty will be in Africa. Moderate poverty, however, will remain high across most regions. Figure 5 illustrates extreme and moderate rural poverty trends from the 1990s with an indicative trajectory to 2025 Africa and South Asia will even see an increase in the numbers of moderately poor. In South Asia, while significant numbers of people are escaping extreme poverty, many are simply being nudged into moderate poverty, with moderate poverty levels likely to further increase over the coming decade. Africa will see extreme poverty plateau, but moderate poverty will increase so that by around 2025, an estimated 534 million people (43% of the continent’s population) will still be living in poverty. With a projected doubling of population in Africa by 2050, the longer-term perspective, unless there is massive economic progress, is particularly concerning. While the situation in East and Southeast Asia appears less dramatic, the very high population there means that even with only 11% living in poverty, this is still over 250 million people.

**Fig. 6:**
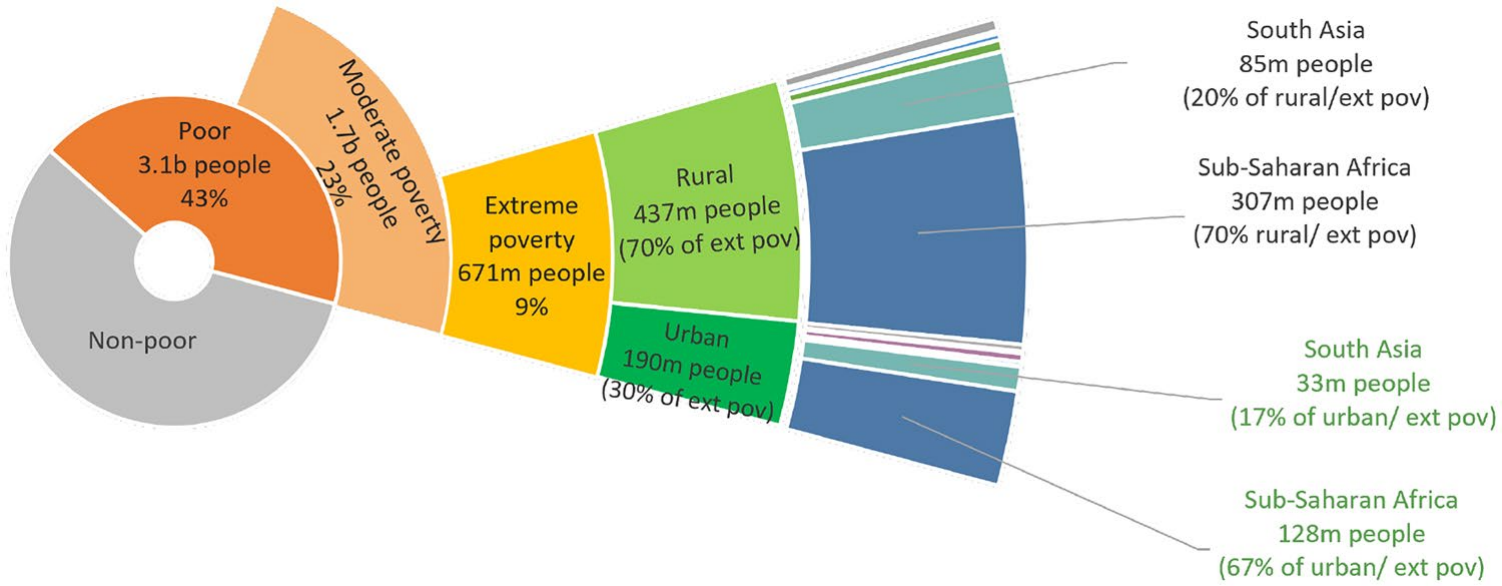
Distribution of extreme rural poverty disaggregated by rural and urban areas and geographic regions Source: Own elaboration using data from Povcal and the World Poverty Clock

**Fig. 7:**
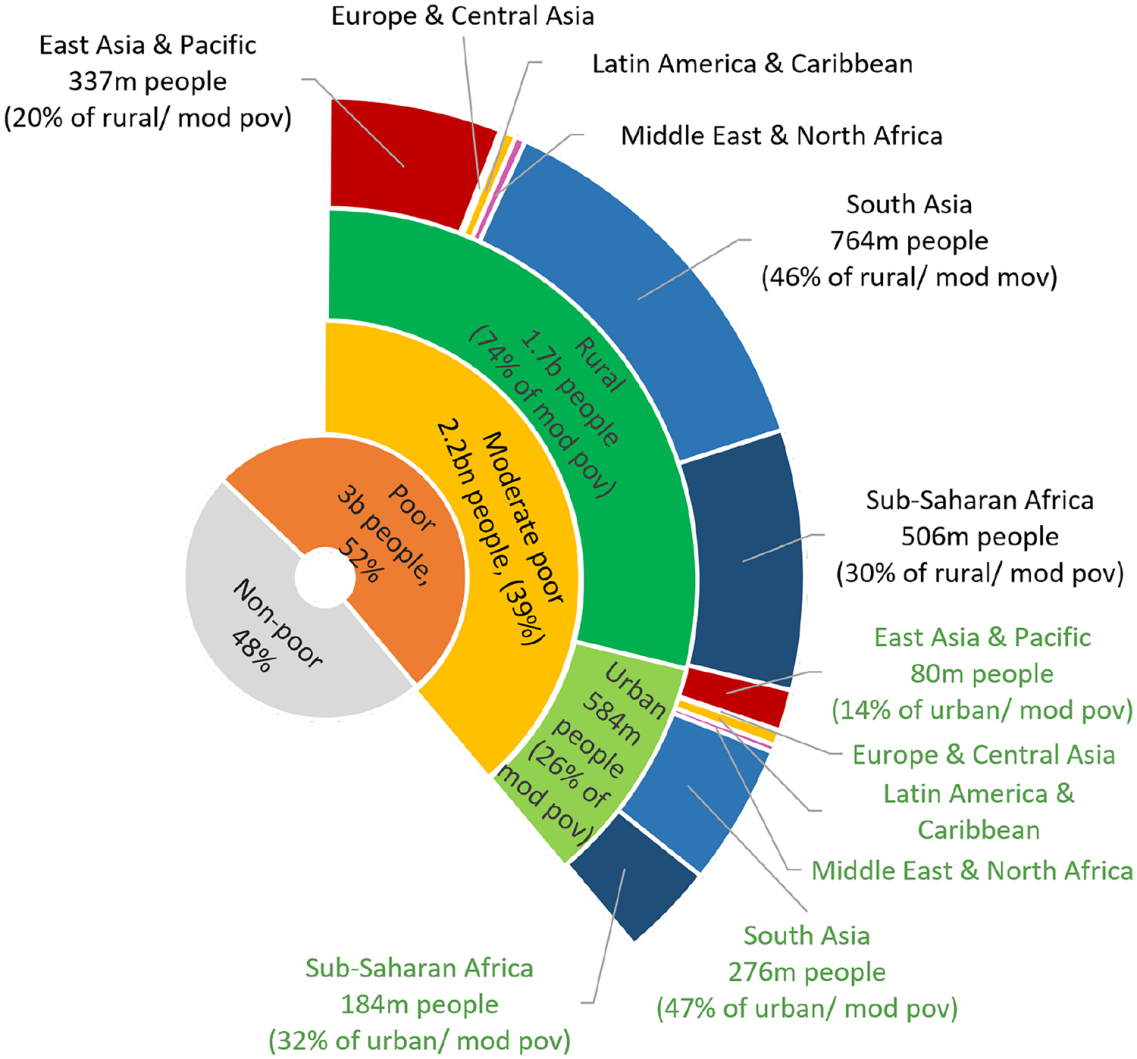
Distribution of moderate rural poverty disaggregated by rural and urban areas and geographic regions. Sources: For moderate poverty FAO (2017a, b), for poverty at $.5.50 per day, Povcal

Figure 6 illustrates the distribution of extreme poverty disaggregated by rural and urban areas and regions, with Fig. 7 showing the same disaggregation for moderate poverty. The reality is that, despite urbanisation, poverty remains concentrated in rural areas and for those working in the agriculture sector. The World Bank estimates that 79% of the world’s extreme poor live in rural areas, even though rural areas comprise only 54% of the global population (World Bank, 2018). In many parts of the world, the majority of the working extreme poor (who live on less than $1.90 a day) work in agriculture: 76% of the working poor in sub-Saharan Africa, 68% in Latin America and the Caribbean, and 56% in Southeast Asia. Agriculture is also the main employer for the moderately poor—61% work in agriculture (Castañeda & Newhouse, 2016). These averages hide great variation across countries and regions. For example, in Ethiopia, 71% of the rural population works in agriculture while across South Asia, this figure is only around one-third of the rural population (see Table 2). Extreme poverty rates in rural zones vary widely as well. In India, 25% of the rural population lives on less than $1.90 a day, compared to 5.5% of the rural population in neighbouring Pakistan (World Bank, 2020e). Projections of changes in agricultural wages suggest that climate change in most low and middle-income countries will cause wages to drop in the period of 2011–2050, with agricultural employment being decreasingly viable path to a stable livelihood (Cui et al., 2018).

Evidence shows a close link between extreme poverty and landlessness in South Asia. In Sub-Saharan Africa, those living in extreme poverty might have access to a very small amount of land but lack other inputs and access to markets (De La O Campos et al., 2018; IFPRI, 2020). A study of 134 countries suggests that poverty rates are higher among overall rural population than among farmers, suggesting that poverty is more likely among landless and non-agricultural rural households (Debucquet & Martin, 2018).

Significantly, while agriculture as a share of GDP has dropped over time, the percent of employment in agriculture and food systems remains high for all countries with high rates of moderate and extreme rural poverty – above 80% for Africa and 50% for South Asia (see Table 2). Structural factors that will affect the future dynamics of food systems and rural wellbeing include the economic scale of GDP relative to the scale of the rural population, population growth rates, the demographics of a large youth population and the scope for economic growth. In this regard, Sub-Saharan Africa faces a particular challenge with a likely doubling of the population amidst more constrained opportunities for broader economic transformation.

Rural inequalities disproportionally affect women and girls. A gender perspective shows that women and girls suffer greater deprivation, fewer economic and life opportunities and higher levels of physical insecurity (Commission on the Status of Women, 2018; FAO, 2011; UN Secretary-General, 2015). Women’s economic empowerment, including, equitable land tenure, access to financial technical services, increased household and community decision making power, overcoming their time poverty, and girls' education are critical foundations for reducing rural poverty and inequality for them and their communities (Andersson Djurfeldt et al., 2018; FAO, 2018, 2020b; FAO et al., 2021; Huyer, 2016; UN Secretary-General, 2019).

The substantial rural youth bulge that will occur over the coming several decades, particularly in Africa, and the employment options in food systems has received much recent attention (IFAD, 2019; IFPRI, 2020; Jayne et al., 2017). By 2030, youth in Africa, Asia, and the Pacific will make up over 77% of the world’s total youth labour force (ILO, 2017). This represents an increase in youth labour by 41.8 million globally. The ‘youth bulge’ in Africa alone constitutes about 55% of the region’s labour force, with 11 million Africans entering the labour force every year. Unfortunately, current estimates show that only 25% of these young women and men will find wage-based employment over the next ten years (Yeboah & Jayne, 2018). Employment alone does not solve issues of poverty among the youth, with 70% of employed youth in Sub-Saharan Africa still suffering from poverty (United Nations, 2018).

The consequences of entrenched rural poverty and inequality are significant. Hundreds of millions of rural people are unable or only marginally able to afford basic levels of healthy food, education, health care, and housing, and are unable to save for emergencies, old age or for other responsibilities and life goals. Further a significant group of people fall in and out of poverty and are highly vulnerable to economic downturns such as that brough on by COVID-19. It is also increasingly well understood that a sense of individual wellbeing along with social cohesion and stability is linked not just to absolute poverty but to relative poverty and feelings of inequality (Wilkinson & Picket, 2010). The scale and future trajectories of rural poverty and inequality, combined with the extent to which rural people earn their livelihoods from food production inextricably links food systems and rural poverty. Rural poverty will not be overcome without making food systems more equitable. Conversely, the poor nutritional and environmental outcomes of current food systems cannot be overcome without tackling rural poverty.

### Nutrition

The world is facing a nutrition crisis – a triple burden of undernutrition, overweight and obesity, and micronutrition deficiencies (FAO et al., 2021). This crisis significantly affects the wellbeing of rural people. The majority of people still suffering hunger, undernutrition and micronutrient deficiencies are rural (ibid). At the same time rural diets are changing towards higher consumption of highly processed low nutrient quality foods with rising levels of overweight and obesity, albeit still lower than in urban populations (Christian & Dake, 2021; Kadiyala et al., 2019; NCD-RisC, 2019; Popkin, 2015; Popkin et al., 2020). These trends indicate that the triple burden will become concentrated in rural populations, with increasing levels of non-communicable disease and reduced earning capacities for those who can least afford it. Furthermore, how the rest of the world chooses to eat in the future will significantly affect rural livelihood opportunities. The consumption of better nutritional quality, safer and higher value food could open up significant economic opportunities for small-scale farmers and enterprises. However, without inclusive policies for food system transformation such opportunities are likely to be captured by larger operators, thereby further marginalising the vast numbers of rural people who depend on food production for their livelihoods.

Many low- and middle-income countries still have child stunting rates in excess of 30%, mostly concentrated in rural areas. Micronutrient deficiencies affect around 2 billion people and obesity levels are rising rapidly (FAO et al., 2021). The distribution of diverse forms of malnutrition across different socio-economic groups and rural and urban populations is complex. However, poverty and under-nutrition are correlated and, as most poor people live in rural areas, under-nutrition in terms of calorie intake is a predominantly rural phenomenon. For example, per capita caloric and protein intake has been falling in rural India, while staying stable in urban India (Deaton & Dreze, 2008). Stunting continues to affect almost half of children under age five living in rural areas in many countries, including those in different vastly different contexts, like the DRC (47%) and India (41%) (WHO, 2020a). Globally children in rural areas are almost twice as likely to experience stunting as children in urban areas (FAO, 2020a). Rural households spend 20–30% less on food than urban households, with more spent on grains (de Bruin et al., 2021).

While overall obesity rates in rural areas are generally still low, obesity rates are growing at alarming rates in rural and urban areas of low and middle-income countries. Rates of diabetes have projected increases of 60–70% for sub-Saharan Africa and South Asia from 2013–2035 (Popkin, 2015). Popkin (2015) shows that in Bangladesh and Ghana, for example, urban obesity rates are twice those in rural areas, and in Ethiopia, the urban obesity rate is five times that of the rural obesity rate. Given that the diets of many rural people are worsening, obesity rates in rural areas are likely to rise further. Despite many rural people still being linked to farming, many are net purchasers of food (FAO, 2017b, pp. 28–29) with purchased food shifting to cheap, highly processed high-calorific and low nutrient quality foods. Additionally, there is evidence of intrahousehold under and over-nutrition, with stunted children and overweight adults.

Women’s empowerment is integral to improving nutritional outcomes of rural households as women strongly influence food decisions and expenditure patterns and enable households to increase their incomes (IFPRI, 2020; Jones et al., 2019).

Poor nutrition strongly affects life opportunities through reduced capacities, reduced earning potential and the costs of nutrition-related diseases (Siddiqui et al., 2020). Beyond individual and household economic impacts, such diseases have astronomical costs for societal health (Bloom et al., 2011). The implications for rural populations, particularly in poorer countries are increased pressures on already limited public finances that potentially negatively affect public investment in rural services and infrastructure. At the global level, ballooning public health costs represents a lost opportunity for investing in productive assets and being able to contribute more to rural economic development.

### Vulnerability

Vulnerability and risk include environmental, climatic, political, social and health shocks that are unexpected and potentially destabilizing to overall wellbeing. The 2020 COVID-19 pandemic and the locust outbreak across East Africa highlighted how vulnerable rural people are to food and economic system shocks. Climate change-driven extreme weather events, natural disasters, and pest and disease outbreaks will increase, possibly dramatically, over the coming three decades. These events will seriously affect vast numbers of rural people and the natural resource base on which they depend, hampering efforts to reduce existing poverty, pushing people back into poverty and creating large scale humanitarian crises (Dasgupta et al., 2014). The number of recorded natural disasters, which are increasingly extreme weather-related, have increased substantially since mid-last century from around 20 per year to 300 to 400 a year. These disasters affect, on average, up to 160 million people per year and over 500 million in some years (IFRC, 2020; WHO, 2020b).

Food systems are highly interconnected with these shocks and vulnerabilities affecting the rural poor. Food consumption and production are major contributors to climate change and resource degradation, for example through land use change and high greenhouse gas emissions and reciprocally food systems are highly vulnerable to climate change-induced shocks (IPCC, 2019; Vermeulen et al., 2012). Rural people are affected in two compounding ways: (1) income loss due to reduced production and rural economy contraction, and (2) increased food prices that they cannot afford which pushes them towards, cheaper and less nutritious diets.

Vulnerability is especially high in fragile states. Definitions of fragile states generally include countries experiencing human-caused crisis and conflict, including violence and institutional failure that leads to instability and human out-migration (World Bank, 2020c). There were 50 fragile states in 2015, according to the OECD. In 2015, these states were home to 1.4 billion people – 20 per cent of the world’s population – and over half are in sub-Saharan Africa. The World Bank estimates suggest that in 2015 roughly one-third of the population in fragile states lived in extreme poverty, with another one-third living in moderate poverty (Collier, 2019). Estimates also show that about half of the population in fragile states live in rural areas (World Bank, 2020g). Rural poverty is the norm in many fragile and conflict states. For example, 89% of DRC’s rural population lives in extreme poverty. Public infrastructure and services in fragile states are often limited or non-existent in rural areas, due to limited resources and political corruption (OECD, 2018). From a global humanitarian perspective, 1.4 billion people represent a massive rural food system challenge in terms of tackling the existing high levels of hunger, averting the risks of larger scale famine, and finding ways to improve the resilience of local food systems in a context of providing food aid.

## Unpacking the dynamics of food systems change for rural wellbeing

We look now at how food systems changes affect the economic opportunities for rural people, in particular the linkages between changing food markets, investment, farm productivity and livelihood options. These dynamics unfold in very different contexts of the political economy, environmental conditions and infrastructure. These differences need to be understood as they have substantial implications for future rural poverty trends, the capacities of states to respond, and the interventions likely to be effective.

### The influence of differing contexts

Rural poverty and food systems are highly influenced by national political economics, natural resources and rural connectivity. These factors will shape the types of food system transformations that could improve rural wellbeing. There are several ways to characterize and assess these contextual factors.

IFAD and FAO (FAO, 2017b; IFAD, 2016, 2019) have assessed rural development and inclusive food systems in relation to a country’s structural and rural transformation, measured respectively by non-agricultural GDP as a proportion of overall GDP and value added per rural worker. Structural transformation involves a shift from primary production-based economies to those with a much higher proportion of manufacturing and services. Associated with this is increased agricultural productivity, rural–urban migration, increased international trade, more capital-intensive forms of production, greater returns to labour and decreasing birth rates. Rural transformation involves increased commercialisation of agriculture, linked with improved agricultural productivity, and is associated with diversifying rural economies and household livelihoods, all underpinned by improved rural infrastructure and services.

This categorisation largely aligns with the World Bank World Development Report (2008) that categorised countries as agriculture-based, transforming (diversifying above) or urbanised (transforming above). These categorisations also align with country income status but provide an additional value of helping to explain underlying economic mechanisms that influence rural development. Rural poverty has dropped most dramatically in fast transforming economies with high economic growth, leading to diversified rural livelihoods (IFAD, 2016). Rural poverty reduction and economic inclusion is a function of both rural economic transformation and wider structural transformation in national economies and how the two processes intertwine (IFAD, 2016); FAO, 2017a).

Alongside a country’s economic context is the degree of social and political stability and the effectiveness of the state. By 2030, over two billion people will live in fragile states, comprising 85% of those in extreme poverty mainly in Africa (OECD, 2020b). Meanwhile those in moderate poverty will be in diversifying middle- income countries. Another factor overlaying country economic context fragility is the nature of the resource base. Thirty five percent of the world’s population (2.1 billion people) live in rangelands that characteristically have poor low productivity natural resources, poor infrastructure and services, and weak market linkages and disproportionally suffer the effects of climate change and resource degradation (Godde et al., 2020; United Nations, n.d.).

A final set of factors to consider are freedom, corruption, and the ease of doing business. Table 3 illustrates the significant variation in these factors across six countries. It is often disadvantaged poor people who are disproportionately negatively affected by constrained freedoms, corruption and difficulties in doing business.

**Table 3:** Indicators of freedom, corruption and business environment for example countries. Sources: ^1^ (Freedom House, 2019) ^2^(Transparency International, 2017) and (Transparency International, 2019) ^3^ (World Bank, 2020a)

	***Countries***					
	**DRC**	**ETH**	**GHA**	**BGL**	**IND**	**PAK**
Freedom status (politics, press, etc.)^1^	Not free	Not free	Free	Partly free	Free	Partly free
Percent of people accessing public services who had to pay a bribe^2^	80	No data	33	No data	69	40
Ease of doing business (0–100 scale)^3^	36	48	60	45	71	61

The underlying structural causes of rural poverty and inequality extend well beyond technical responses, no matter how important initiatives are for agricultural productivity, rural infrastructure, access to finance or markets. This political economic context shapes the nature and functioning of local, national and regional food systems. It also sets the boundaries around the extent to which a transformation of food systems can drive a reduction in rural poverty and inequality and the capacity of national governments to respond.

The 2008 World Development Report on agriculture (World Bank, 2008) noted that a lack of attention to the political economics of agriculture and rural development was a key reason for not implementing reforms recommended 20 years earlier for an agricultural (food systems) led approach to tackling rural poverty. Meanwhile the African Agriculture Status Report (AGRA, 2018) notes the critical need for political will to drive the investments and reforms needed for rural transformation, which are largely well understood but not acted upon.

### The market revolution

Reardon et al (2019) refer to a “quiet revolution” in food markets in low and middle-income countries. This is the rapid growth of micro- small- and medium-scale enterprises operating in a transitional food market structure, driven by urbanisation, increasing wealth and nutritional changes (FAO, 2017b, p. 13). Transitional markets function in the middle space between traditional, informal markets (with no contracts and ‘spot market’ cash-based transaction) and modern markets, which include long supply chains (rural to urban and international), consolidation and concentration of capital and standards (the supermarket model) (Reardon et al., 2019).

The scale of this market change over the last 20 to 30 years has been profound and will continue over coming decades (FAO, 2017b, p. 28). Haggblade et al. (2010) estimate an increased in flow of food products from rural to urban areas at 600–800% from the 1980s to 2010 for Africa while Reardon and Timmer (2014) have it at approximately 1000% in Southeast Asia over the same period. These market changes are underpinned by deep structural shifts in procurement, retailing, value chain coordination, ownership, and power relations between larger and smaller scale operators in the food system. The central observation of Reardon et al. (2019) is that transitional markets dominate in the food systems of low- and middle-income countries and are likely to do so for the foreseeable future. Because of large rural populations in many areas traditional markets will also co-exist with transitional markets well into the future.

These market changes have occurred through large-scale endogenous processes and often despite the constraints of transport, finance and market distortions. It has been driven largely by the domestic private sector, arguably government or donor-driven market development initiative have only had a minimal influence on the overall scale of this change. During the same period, global trade in food has also increased (FAOSTAT), with markets becoming more liberalised and competitive. Low- and middle-income countries have seen a substantial increase in the consumption of highly processed food (Baker & Friel, 2016; Reardon et al., 2021), even in poor rural areas, leading to increased domestic food processing.

The economic benefits from this food market growth have been far from universal and equitable. The urban demand for food is more easily met by production regions that have good infrastructure, market access and production conditions, and by farmers who have better assets in terms land size, access to capital and other services, and skills (Fan & Rue, 2020; Rapsomanikis, 2016). As discussed in Sect. 3, for many countries the bulk of food is produced by a smaller group of larger (but still small-scale) farmers. The need for bulk quantities, improved standards, economies of scale, and more sophisticated production systems for non-staples further constrains who can benefit from these growing markets (Fan & Rue, 2020). There is also considerable competition with food imports, which can often fill demand more cheaply than domestic production, particularly in Africa as illustrated by the rising rate of food imports (FAOSTAT).

The transitional and informal nature of much employment in the agriculture and food sector, particularly where there is growing youth unemployment, also create the potential for poor working conditions and exploitation. The reality would seem that on their own, these growing markets will not create a sufficient scale of inclusive economic development to reduce inequality and substantially benefit poorer and more marginalised rural groups being left behind.

### Emergent investment

The market revolution described above and the overall increasing global demand for food is driving emergent investment in food systems, both foreign and domestic. Many lower and middle-income countries are seeing growth and domestic investments in the food and agriculture sectors, much at a small to medium scale, for example Jayne et al. (2021) reports agricultural growth in sub-Saharan Africa at a high 4.3 percent since 2000. These private domestic investments in food and agriculture are far larger than those of foreign private investors, national governments and development agencies (Lowder et al., 2015). There is growing evidence of salaried urban elites making substantial investments back into agriculture as “emergent farmers” (Jayne et al., 2016a, b) and food sector entrepreneurs. This investment offers both opportunities and risks. It enables countries to meet growing urban food demand and drives growth of the agrifood sector. However, there has been limited domestic application of principles of responsible agriculture investment, such as the CFS-RAI. This increases the risk of domestic land grabbing, poor environmental practices and poor labour conditions.

Jayne et al. (2015) shows that medium-scale farms are the fastest growing segment of the family farm sector in Sub-Saharan Africa, controlling more land than large-scale farms. However, there is evidence that much of this growth comes from investment by urban and rural elites (emergent farmers) and not from existing small-scale farmers graduating to become larger and commercially viable. While such investment is needed, it has implications for the transformation of small-scale agriculture. If new market opportunities in agriculture are being taken up on a significant scale by emergent investors, it potentially crowds out opportunities for existing small-holders and undermines the development narrative of tackling poverty by helping to connect small-scale farmers to markets. There are also arguments that this emergent investment could occur in inclusive ways that are synergistic between emergent and traditional small–small scale farmers.

As yet there is insufficient data to show overall trends and effects of this emergent investment and its impact on commercialisation of existing small-scale farmers. It is a trend that needs careful attention in order to understand the dynamics of transforming small-scale agriculture. Many small-scale farmers and aspiring agri-food sector entrepreneurs lack sufficient access to capital to run and expand their enterprises. This constrains a more inclusive development of the sector as emerging opportunities can be captured more easily by elites who have capital and income, often from outside the sector, and who can also afford to take risks in shifting to new markets or production systems. This particularly constrains rural youth and women from taking up agri-food sector opportunities.

While domestic private sector investment into the food and agriculture sector is growing this is not matched by public sector investment or foreign direct investment. For the period 2013–16, G20 gross fixed capital formation in agriculture fell to 0.2 percent compared to 4.5 percent for the period 2002–2012. From a high in 2007 of over nine billion USD, foreign direct investment in agriculture has dropped substantially over the last decade to below two billion in 2017 (FAOSTAT). From 2001–2016, the Agricultural Orientation Index for Government Expenditures (AOI) showed a decline from 0.42 to 0.26 indicating a general relative decline in public investment in agriculture for all countries (FAOSTAT). Middle- and low -income countries invest relatively less in agriculture than high income countries, despite it contributing more to GDP and employment and their potential for agri-food sector expansion. The (2020) CERES report estimates that to end hunger would require an additional public investment from donors and national governments of USD 33 billion per year, which would also spur an additional annual investment of USD52 billion from the private sector.

### Farm productivity, profitability and production diversity

Rural transformation and wellbeing depend on farm productivity and profitability. However, in many parts of the world, particularly Africa, and for many poorer rural households there remains a substantial yield gap (Giller, 2020; Rong et al., 2021). The negative effect on profitability is compounded by high input costs and often low farm-gate prices. The Green Revolution and agricultural development at the end of last century focused almost exclusively on increasing the yield of staples, largely through improved varieties and the application of external inputs (Ameen & Raza, 2018). The world now faces an evolving and increasingly complex set of agricultural production challenges (Calicioglu et al., 2019; FAO, 2017a). Poor farm productivity still afflicts much of the more impoverished parts of the world. This challenge is being compounded by the impacts of soil and water degradation, climate change induced weather extremes and increased risk of pest and disease outbreaks. Further, a change is needed in the balance of production to increase nutrient rich crops such as fruit and vegetables relative to energy dense crops such as starchy staples for improved human nutrition (Fanzo et al., 2020). This change needs to occur in tandem with forms of agriculture that mitigate climate change (Lynch et al., 2021). The effects of environmentally unsustainable food systems impact far more dramatically on rural populations than on urban consumers.

These dynamics are a further driver of increasing inequality in rural wellbeing (UNDESA, 2021). In general, poor productivity, resource degradation and climate impacts are much worse in marginal areas where there are higher levels of poverty. Poor households and communities are less able to cope with the shocks of climate extremes or disease outbreaks (UNCCD, 2019). A nutrient rich diet is more expensive, which the poorest and most malnourished people are not able to afford (Hirvonen et al., 2020). Further, shifting to more resource efficient, climate smart and nutrient rich cropping systems requires access to technologies, capital, management skill, market linkages and ability to absorb risk that is often difficult or impossible for poorer farming households.

Whilst a large body of evidence exists on the critical role gender dynamics play in small-scale agriculture and rural poverty (Huyer, 2016; FAO, 2018), there is a need to better understand how those dynamics are changing at farm level and elsewhere within the food system, and how these dynamics differ across contexts. As a recent FAO report (FAO, 2017b, p. 88) notes, the observed ‘feminization of agriculture,’ exemplified by the increased female share in agricultural employment (almost 50% in some regions) is occurring for many reasons. In some areas of India, for example, men are moving out of agriculture into higher-paying sectors as rural development occurs (Pingali et al., 2019). In many other countries, especially in sub-Saharan Africa, out-migration of men, to cities or other countries, has left women taking on new roles as primary food producers (FAO, 2017b).

Even as women’s role in more formalized agricultural activities seems to be increasing, their factor productivity (yield, return on investment in inputs, etc.) remain far lower than men’s. The *Enabling the Business of Agriculture* report (World Bank Group, 2017) describes how dramatic the ‘gender productivity gap’ can be, as in Niger where it is estimated to be 66%. In most countries the gap is estimated to be around 20–30%. Many reasons interact, including time pressures due to dual responsibilities of care and farming, limited decision-making power, inadequate land tenure rights, and low access to finance. These differences have social and economic consequences for women farmers, while also significantly impacting the wider economy. Some sources suggest that equalizing this gap could boost agricultural output and decrease global undernourishment by up to 17 percent (Doss et al., 2018; Oxfam, 2017).

### Livelihood options

These dynamics of markets, investment and farm productivity have significant implications for the future livelihood options of rural households. The implication is that farming on its own has limited potential to overcome the scale of moderate and extreme rural poverty still being experienced by many countries, particularly where there is a growing population of youth and fragmentation of land (Giller et al., 2021). Future strategies for overcoming rural poverty will need to look to a more diversified and integrated set of livelihood options. These include enterprise development and employment in the off-farm economy, particularly in the agri-food sector (Vos & Cattaneo, 2021), remittances, and improved access to social protection. Such diversification will need to include farming households integrating farming with other livelihood options, as many are already doing, as well as shifting out of farming to alternative employment and enterprise opportunities (Fan & Rue, 2020).

Rural poverty reduction requires attention for expanding opportunities across the entire agri-food economy of rural areas. In particular, efforts are needed to ensure that value is added to farm production in rural areas to complement farming in attracting financial returns from the food sector into the rural economy, helping to generate rural employment and economic development (Haggblade et al., 2010; Vos & Cattaneo, 2021).

However, alternative livelihood options are not immediately or easily available to many poorer farming households. Many will still depend on farming for at least part of their livelihood for the foreseeable future, despite meagre returns. Countries will need transition strategies to support small-scale farmers who in the shorter-term are caught in a poverty trap, while working towards longer term viable livelihood options. These strategies should include ensuring poor and marginalised groups are not dispossessed of what limited natural resources they have, improving the performance of semi-subsistence agriculture, providing support to access off-farm livelihood opportunities, improving flows of remittances, and targeted social protection schemes.

At the same time, ensuring a viable commercial small-scale agriculture sector remains a critical foundation for rural economic development (Mellor, 2017; Mellor & Malik, 2017). Viable small-scale agriculture is needed to meet the food demands of growing urban populations and to attract profits back to rural areas that can drive growth of the wider rural economy. However, in the long run, only a minority of better endowed small-scale farmers are likely to make the transition to commercial viability that provides a decent income from farming. The proportion of small-scale farmers making this transition will depend strongly on local circumstances and the nature of agricultural and wider food systems policies.

The most obvious alternative livelihood strategy for farming households is participation in the midstream of the food system between production and consumption. Midstream economic activities include input supply, mechanisation services, advisory services, trading, processing, marketing and food services. The informal and semi-formal nature of this sector offers a wider range of opportunities that can be taken up without necessarily high-level skills and/or capital investment. As outlined in Sect. 5.2 above micro, small- and medium-scale enterprises have expanded rapidly over recent decades, and as noted in Sect. 3 farming households are already diversifying into employment and enterprise in this midstream.

However, the full extent of economic value and employment opportunities in the midstream is not well quantified. Caution is also needed about the extent of the benefits from growth in the food systems economy, as employment conditions in the informal food economy and on-farm labouring are often poor or even exploitative, particularly where there is an oversupply of labour. Off-farm employment will not necessarily lift people above the poverty line or deliver them a decent income unless policies and standards are in place to ensure fair employment conditions. Further, over time the modernisation of food markets, as ever greater economies of scale and efficiency are sought, drives towards a mechanised capital-intensive state with reduced labour demand. An important policy challenge for low- and middle- income economies will be to develop policies for the midstream of the food system that optimise the value creation and employment in the midstream, while also meeting the need for upgrading value chains to meet demands for food quality and safety, scales of efficiency and competitiveness.

In rural people’s livelihood mix, remittances are also important to consider and can be a substantial contribution to household income as well as providing a foundation for investment in farm and off-farm enterprises (Gelb et al., 2021; World Bank, 2019). The scale of remittances varies significantly between countries. In many rural or fragile countries, like the DRC and Ethiopia, average per capita annual remittances are quite small (22 and 4 USD respectively). In other countries, including Ghana and Pakistan (118 and 100 USD respectively), per capita remittances have the potential to greatly impact economic realities if they are flowing to rural areas (World Bank, 2020c).

### The challenge of leaving no-one behind

Realism is needed about the scope for lifting the large numbers of rural people who are being left behind out of poverty through economic opportunity alone. Extreme poverty is becoming increasingly concentrated in a limited number of countries with conflict and/or fragile states (World Bank, 2021, p. 21), where the food sector and wider economic growth is limited. However, virtually all low and middle-income countries have significant numbers of excluded, vulnerable and extremely poor people living in rural areas (World Bank, 2020f). Many of these people are in areas of poor resources and infrastructure (Ahmadzai et al., 2021). Further, many poorer groups are disadvantaged and marginalised in ways that exclude them from easily taking up economic opportunities. This is critical to recognise in understanding the scope and constraints for agricultural development and food market growth to be a driver of poverty reduction in these areas. Further, in such areas livelihood diversification from off-farm or non-agricultural employment and remittances is often lower. This compounds the development challenges for these areas, making public investments in development and social protection critical for tackling poverty, malnutrition and vulnerability.

## Linking food systems transformation and rural wellbeing – policy implications

The above analysis illustrates how the underlying dynamics of rural poverty, inequality and vulnerability are changing, with profound implications for future policy making. Historically, comparatively simple approaches of increasing agricultural productivity combined with rapid growth in the wider economy lifted billions of rural people over the poverty line (World Bank, 2008). Today however, the challenges are more complex (Christiaensen & Demery, 2018; Christiaensen & Martin, 2018). Extreme rural poverty is increasingly concentrated within certain groups and geographies characterised by deep structural barriers to economic development. Rural inequality (moderate poverty) is embedded in a wider global phenomenon of rising inequalities and unequal economic progress (World Bank, 2020f). Resource degradation, the impacts of climate change, emerging pest and disease risks and changing geopolitics are creating new vulnerabilities, potentially affecting large groups of rural people. Critically, the models of food production and consumption in which past approaches to agricultural and rural development have been embedded have given rise to huge environmental and human health externalities—prompting the UN Secretary General to call for a food systems transformation (United Nations, 2021).

### Three policy priorities

We argue that improving the wellbeing of future generations of rural people will require policy innovation across three broad areas. These are: (1) changing incentive structures to tackle the negative market externalities of the food system at large, (2) investments that enable rural economies to capture greater value from the food system, and (3) improved social protection and humanitarian relief schemes that support those in crisis or being left behind. If these are taken seriously, they would require profound changes in regulations, taxation arrangements, trade regimes, subsidies and patterns of public investment.

There is no shortage of general recommendations about what is needed to improve rural development, small-scale agriculture and the rural agri-food sector (AGRA, 2018; FAO, 2017b; IFAD, 2016; IFPRI, 2020; Woodhill et al., 2020). The menu is well-established: infrastructure, better public services, access to financial services, improving the functioning of input and output markets, private sector engagement, research and development, effective producer organisations, land tenure reform, women and indigenous people’s empowerment, territorial approaches and social safety nets. However, the necessary scale of investments is not being made by national governments or the international donor community (David Laborde et al., 2020). Further, the incentive structures for how food systems currently function do not drive the scale and type of private sector investments needed for sustainable and inclusive outcomes (World Economic Forum and McKinsey and Company, 2020).

The growing realisation of how detrimental current food systems are for nutrition, public health, the environment and climate change (illustrated by the engagement and narratives around the UN Food Systems Summit) potentially opens the policy space for connecting the agenda of rural poverty into this wider agenda of food systems transformation. It is estimated that externality costs of the food system in terms of poor health, resource degradation, climate change and poverty add up to 2 trillion more than the estimated 10 trillion annual GDP of the food system (FOLU, 2019). For society at large, over the longer term, reforming the market incentives that drive this situation represents the potential for a significant return. However, as discussed below reforming the underlying incentive structures of food systems is a difficult governance challenge.

In terms of investments that enable rural economies to capture more value from the food system, there is a need to complement agricultural production with much greater attention for upgrading the midstream of food systems and expanding employment opportunities in agri-food processing, distribution and services sectors (AGRA, 2019; UNDESA, 2021). Polices are needed that enable and support rural people to be entrepreneurial and establish and run successful micro-, small- and medium scale businesses. The challenge here is to enable investment by rural people and for returns to be fed back into the wider rural economy, thus driving further economic and employment opportunity. Creating such an inclusive rural food economy will require substantial policy innovation to avoid the agri-food sector consolidation that has been seen in high-income economies with returns being captured predominantly by larger firms and investors.

To overcome continuing levels of extreme poverty, avoid growing inequalities and enhance resilience, society will need to find ways of filling the vast social protection gap that exists in low and middle-income countries. Just under half the global population is covered by at least one social protection benefit, in Africa it is less than 20% and in Asia and the Pacific under 40% (World Bank, 2020g). Even by optimising enterprise and employment opportunities in the food system and wider rural economy, high levels of underemployment will remain. Hundreds of millions of farming households will remain in a transitional or “hanging-in” state, not able to earn a decent living from their current livelihood activities but also unable to move into new opportunities. Meanwhile climate change and other shocks will exacerbate crisis situations. Further, society is seeing a general trend towards automation, including in the agri-food sector putting a downward pressure on employment opportunities. All these factors call for a deeper look at social protection policy. However, to be affordable and effective, social protection policy innovation is needed around insurance mechanisms and forms of ‘productive’ social protection that support economic integration with building household/community resilience. There may be a case for exploring the options for a universal basic income (Standing, 2021). As this article has outlined, a substantial scale of rural poverty and vulnerability continues in fragile states and low-income countries, where the state has limited capacity to adequately respond. The implications is a need for the international community to step up support to avoid the levels of social and political strife, humanitarian crises, and migration that will be inevitable if rural people are left in a state of poverty and vulnerability.

## Conclusion

Our analysis suggests that the future wellbeing of large numbers of rural people are at significant risk. This assessment comes from taking a broader view of wellbeing than just levels extreme poverty and hunger/child stunting. The numbers of rural people whose wellbeing is at risk increases dramatically if all forms of malnutrition, all levels of poverty and inequality, and vulnerability, particularly related to climate change, are considered. This provides a much more sobering perspective on the degree of progress in rural development over recent decades and on the scale of future challenges, than by looking only at the reduction in levels of extreme poverty. The analysis also shows how interconnected rural wellbeing is to food systems, both in terms of risks and opportunities for improvement. We also highlight how rural wellbeing and opportunities for improvement, differs across different geographic and political economic contexts with different countries and regions having very different dynamics and prospects. We argue that taking a food systems perspective means that the role of small-scale agriculture needs to be seen in a wider context and against the significant structural changes occurring in food markets and rural economies. In particular, the challenge for the very large number of very small-scale farmers who are unable to make a living income from farming needs much more attention with solutions that go beyond agriculture alone. This situation needs to be understood in relation to significant diversification of rural household incomes which is occurring in most rural areas.

Rural wellbeing into the future will depend on bringing about significant structural changes in food markets, economic policy and patterns of public and private investment. The challenges are only partly technical, the root causes and effective solutions of rural poverty and inequality are tied to incentives embedded within the political economic and power structures of the wider food system. A food systems transformation that will be more equitable for rural people calls for processes of dialogue, engagement, and empowerment that drives the institutional and political innovation needed to reshape societal understanding, power dynamics and the political will for change.

To drive an inclusive food systems transformation that substantially benefits rural people policy makers will need to give greater attention to the processes of stakeholder engagement, and data gathering and analysis, needed to support such deeper structural and political economic change. Enhanced processes of foresight and stakeholder dialogue and societal learning at local and national scales can be part of the solution. These processes will need to be underpinned and informed by a better analysis and synthesis of the dynamics of rural poverty and food systems change. A food systems transformation that creates rural wellbeing will require new forms of national and local engagement, analysis, dialogue, coalition building and leadership. This can be strengthened through global public good investments which better balance support for human capacity development and institutional reform with support for technical solutions.
